# Influence of Reheating Methods and Frozen Storage on Physicochemical Characteristics and Warmed-Over Flavor of Nutmeg Extract-Enriched Precooked Beef Meatballs

**DOI:** 10.3390/antiox9080670

**Published:** 2020-07-27

**Authors:** Rashida Parvin, Md. Ashrafuzzaman Zahid, Jin-Kyu Seo, Junyoung Park, Jonghyun Ko, Han-Sul Yang

**Affiliations:** 1 Division of Applied Life Science (BK21 plus), Gyeongsang National University, Jinju-daero, Jinju, Gyeongnam 52828, Korea; rakhi@gnu.ac.kr (R.P.); zahid@gnu.ac.kr (M.A.Z.); jkseo@gnu.ac.kr (J.-K.S.); jypark@gnu.ac.kr (J.P.); jhko@gnu.ac.kr (J.K.); 2 Institute of Agriculture and Life Science, Gyeongsang National University, Jinju-daero, Jinju, Gyeongnam 52828, Korea

**Keywords:** precooked beef meatballs, nutmeg extract, reheating, oxidative stability, warmed-over flavor

## Abstract

The effects of convection-oven precooking, frozen storage (−18 °C/ two months) and four different reheating methods—namely, boiling, pan-roasting, convection oven and microwave oven on pH, color, texture, antioxidant activity and warmed-over flavor of beef meatballs were investigated. In this study, four kinds of beef meatballs were prepared: with added butylated hydroxyl toluene (0.02% BHT, M1); with nutmeg extract (0.02%, M2); with nutmeg powder (0.02%, M3) and control (no antioxidant). Addition of (0.02%) nutmeg extracts in beef meatballs M2 resulted in a significant (*p* < 0.05) decrease in lipid and protein oxidation, hardness and gumminess values after convection oven precooking. Again, M2 reheated by microwave oven significantly (*p* < 0.05) reduced cooking loss, gumminess, springiness, rancid flavor, saltiness and burnt taste and increased oxidative stability, redness and adhesiveness with the chewiness intensity and overall acceptability compared to control, M1 and M3. Conclusively, the addition of nutmeg extracts (0.02%) as a natural plant antioxidant to precooked beef meatballs can result in reduced lipid and protein oxidation levels, stabilized color and texture values and improved overall acceptance after reheated by microwave oven during two months of frozen storage.

## 1. Introduction

Processed meat in ready-to-eat (RTE) forms is demanded by modern consumers to save time and effort in their busy lives. Beef meatballs are prepared by adding flour, fat, water and spices with ground beef and processed by precooking, cold storage (refrigeration and frozen) and reheating to make suitable for intake [1] All precooked products demand time-specific internal reheating before intake to make food safe and prolong shelf life without affecting nutritional qualities of today’s market. Meat is rich in polyunsaturated fatty acids, and processing of meat by cooking, reheating and storage disrupts muscle cell membrane and initiates lipid oxidation which deteriorates the quality of attributes such as color, flavor and odor of meat [2] The application of heating or cooking is used to make the food soft and easily digestible. Different heating times and temperatures are used for different cooking system like boiling, grilling, roasting, steaming, pan-frying, microwave-oven cooking and convection-oven cooking. Food flavor and eating quality are expressively influenced by the type and quantity of fundamental components like lipids, proteins and carbohydrates of meat products [3]. Post-preparations, including handling, cooking method and holding period before serving, characteristically determine the sensory quality and acceptability of the meat product for ingestion [4]. Hard texture with low moisture is a common phenomenon for repeatedly heated food items [5]. Customer approval for cooked meatballs is mainly decided by the flavor, which is influenced by different cooking methods and storage conditions. The flavor of cooked meat is malformed by developing diverse aromatic compounds due to oxidation of lipids, proteins and flavor compounds and Maillard reactions [6]. Lipids and proteins are easily oxidizable biomolecules of meat products that generate toxic compounds and decrease product shelf life. Accumulation of salt during meat processing is a causal factor of oxidation [7]. Rapid lipid oxidation generates an off-flavor known as warmed-over flavor (WOF) in precooked meat products after cold storage and diminishes product quality [8].

Antioxidants are preservatives used to delay or restrain the oxidation process, develop color stability and expand shelf life during the processing of meat products [9]. Synthetic antioxidant like BHT (E321) has been widely used in meat industry to prevent oxidation, stabilize color and flavor, and enhance product keeping quality. However, only 0.02% of BHT (E321) is permitted in meat products, and their application has to be declared on the label of packaged meat itself [10]. Carcinogenic effects of synthetic antioxidants and consumer preference for natural products to food additives have resulted in increased interest in the use and research on plant origin natural antioxidants [11]. Nutmeg seed (*Myristica fragrans* Houtt.) has been familiar to most countries as common cooking spices, used as a culinary adjunct in meat and imparts pleasing flavor of the product, also exhibit α-pinene, β-pinene, p-cymene, β-caryophyllene and carvacrol, which are acting as antioxidants [12]. The main flavor compounds of nutmeg are oleoresins which can withstand the high-temperature processing [13] and convey a pleasing aroma during the making of meat and vegetable dishes. The rancid effect of lipid oxidation is effectively minimized by using nutmeg extract as per the literature review [14], which is a beneficiary advantage for our study.

As followed the lifestyle of modern people, they like ready to eat fast food. The precooked meat product which stored in cold condition develops an unpleasant flavor after reheating, demand the anticipation of oxidation and off-flavor formation by modifying meat product formulation and processing techniques. Keeping this in view, BHT (E321), nutmeg extract and nutmeg powder were added to beef meatballs, and the comparison of the effects of these antioxidants on lipid and protein oxidation, color and sensory attributes in cooked beef meatballs was studied. Again, the effect of synthetic antioxidants, different cooking methods and single reheating method on refrigerated meat were previously studied by many researchers [15,16]. However, it is necessary to study the effect of natural antioxidant, single precooking method, different reheating methods and frozen storage on the physicochemical and sensory qualities of meat products and suggest a particular cooking method and natural antioxidants for good quality meat product while reheating at commercial level.

Therefore, the objectives of this study were to evaluate the efficacy of nutmeg extract as natural additives on precooked beef meatballs under different reheating condition. This efficacy was compared with an artificial antioxidant during frozen storage.

## 2. Materials and Methods

### 2.1. Chemicals

All required chemical reagents and solvents used for this study were analytical or chromatography grade and collected from Sigma-Aldrich (St. Louise, MO, USA). Distilled and deionized water used were purified by Milli-Q system (Millipore Corp., Bedford, MA, USA).

### 2.2. Preparation of Nutmeg Powder and Extract

Four bags (300 g weight each) of whole matured, dried and healthy *Myristica fragrans* Houtt. nutmeg seeds were purchased two times from herbal medicine stores in Jinju, Korea to make nutmeg powder and extract in this study. Dried seeds were carefully checked and ground into a fine powder using a Waring blender (12,000 rpm) and sieving machine and the fine powder was kept in an airtight non-reactive box at the cool place until use. Ethanolic extraction of dried nutmegs was done by mixing 150 g of dried nutmeg powder with 70% ethanol of 1:10 *(w/v)* ratio at 60 °C for 15 min. After extraction, the remaining materials were strained using Whatman No. 4 filter papers. The strained solution was evaporated to dry using a vacuum rotary evaporator (Scietek, MODEL: RE 300) at 70 °C and freeze-dried for 48 h to get dry nutmeg extract. Then, the extract was preserved in a plastic dish covered with a lid at −20 °C until use.

### 2.3. UPLC-MS/MS Analysis for Qualitative Bioactive Compounds of Nutmeg Extract

The bioactive compounds of nutmeg extract by 70% ethanol were analyzed with a UPLC system (Waters, Milford, MA, USA). The samples were injected into an Acquity UPLC BEH C_18_ column (100 × 2.1 mm, 1.7 μm; Waters) balanced with water containing 0.1% formic acid and eluted in a gradient with acetonitrile (ACN) containing 0.1% formic acid at a flow rate of 0.35 mL/min for 10 min. The eluted metabolites were analyzed by a Q-TOF MS (Waters) in electrospray ionization (ESI)-positive mode. The capillary and sampling cones voltages were set at 3 kV and 30 V, respectively. The temperatures of the source and desolvation were set at 100 °C and 400 °C, respectively, and the desolvation gas flow rate was 800 L/h. TOF MS data were collected in the *m/*z** 50–1500 range with a scan time of 0.2 s. Lock spray with leucine–enkephalin (556.2771 Da) was used at a flow rate of 5 μL/min and a frequency of 10 s to ensure accuracy and reproducibility for all analyses. For quality control (QC), a mixture of all samples was injected after every 10 samples. The MS/MS spectra of the metabolites were collected in the *m/*z** 50–1500 by a collision energy ramp from 10 to 30 eV. All MS data obtained by Mass Lynx software (Waters), including retention time, *m/*z** and ion intensity, were extracted with Marker Lynx software (Waters).

### 2.4. Formulation and Preparation of Meatballs

Fresh lean beef (Semimembranosus muscle, SM) and backfat were procured separately from a local meat processor, Jinju, Korea and stored at −18 °C until use. Thawed meat of large size and animal fat were minced firstly using a 3-mm plate and a combination of ground meat and fat were divided into four batches. Twenty grams each meatballs were formulated as follows the basic ingredients (beef meat 70%, beef fat 20%, iced water 4%, corn starch 5% and salt 1.5%) and 0.02% antioxidant like BHT for M1, 0.02% nutmeg extract for M2, 0.02% nutmeg powder for M3 and an antioxidant free control group. The mixture of basic ingredients was transferred to a mixer and mixed with each antioxidant separately for 5 min to make a homogenous batter for different treatments. Then, each meatballs (approximately 30 mm diameter, 20 g each) was shaped properly by hand and arranged them separately for each treatment per each batch.

### 2.5. Cooking Methods

Convection oven was used primarily to cook all samples of each treatment independently at 120 °C for 15 min to make precooked meatballs. After cooking, the meatballs were cooled quickly at room temperature, and samples from each treatment were randomly collected for analysis of physicochemical characteristics of precooked meatballs. Next, meatballs of all treatments were aerobically packed in high density polyethylene bags separately with proper sealing and stored at −18 °C frozen storage for 1 and 2 months separately.

**Figure 1:**
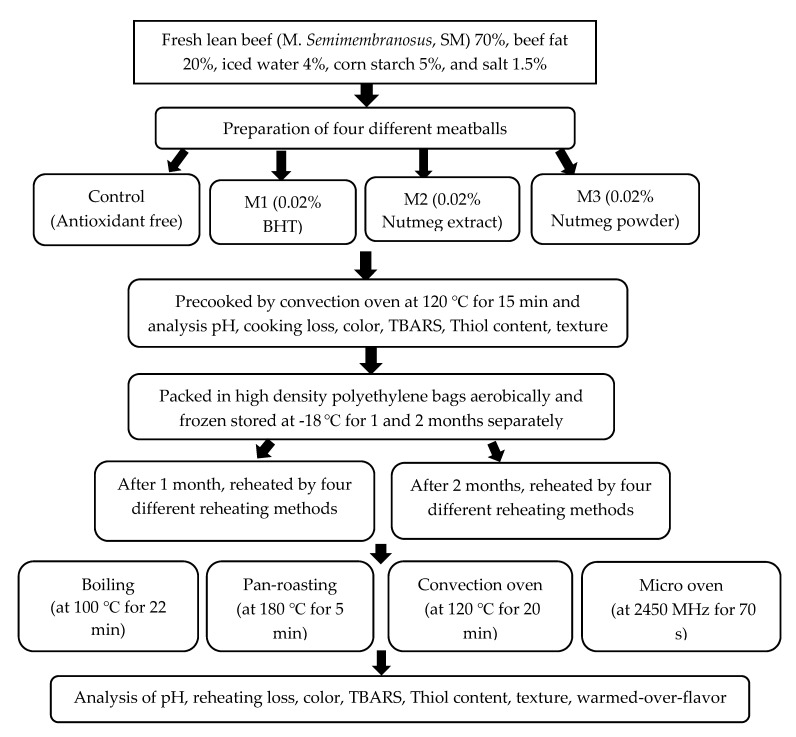
Diagram of used experimental design.

After 1 and 2 months of freezing, samples were taken for four cooking methods; boiling (at 100 °C for 22 min), pan-roasting (at 180 °C for 5 min without using any cooking oil), convection oven (at 120 °C for 20 min) and microwave oven (Model RE-M400, Samsung Electronics Co., Ltd., Suwon, Korea, at full power 700 W, operating at 2450 MHz for 70 s) were used for reheating the meatballs samples. The combination of cooking time and internal temperature of 75 °C were preliminarily settled by using a thermocouple (ME32, Metex, Seoul, Korea) to determine the end point of cooking. An overview of the used experimental design is shown in Figure 1.

### 2.6. Analytical Methods

#### 2.6.1. pH

For pH test, 3 g minced sample was homogenized with 27 mL distilled water for 30 s using a Polytron homogenizer (T25 basic, IKA, Selangor, Malaysia). Then, the slurry was kept to set at room temperature. The pH meter (MP230, Mettler Toledo, Greifensee, Switzerland) connected with an electrode probe (Mettler-Toledo, Inlab Semi Micro Electrode, Greifensee, Switzerland) was calibrated using pH 7.00, 4.01, and 9.21 buffer solutions, and readings were taken two times for each treatment.

#### 2.6.2. Cooking Loss and Reheating Loss

Cooking loss of all precooked meatballs was determined by calculating the weight difference between before and after cooking of meatballs. Reheating loss of all reheated meatballs for each reheating method was assessed by calculating the weight difference between before and after reheating of precooked meatballs as follows:

(1) $$Cooking\;loss\%=\frac{wt.\;of\;raw\;meatball-wt.\;of\;cooked\;meatball\;}{wt.\;of\;raw\;meatball}\times 100$$

(2) $$Reheating\;loss\%=\frac{wt.\;of\;precooked\;meatball-wt.\;of\;reheated\;precooked\;meatball\;}{wt.\;of\;precooked\;meatball}\times 100$$

#### 2.6.3. Thiobarbituric Acid-Reactive Substances (TBARS)

The modified extraction method of TBARS suggested by [17] and the TBA distillation procedure given by [18] were used for understand the status of lipid oxidation in the cooked beef meatballs. The values were expressed by mg of malondialdehyde (MDA) for each kg of sample. For this assay, 3 g of ground samples were homogenized at high speed with 27 mL 3.86% perchloric acid using a digital homogenizer for 20 s and kept for 1 h at low temperature to settle down properly. Next, the mixture was passed through a centrifugation machine at 2000 rpm for 10 min and clarified using Whatman no.1 filter paper to separate the lipid part from other macronutrients. By using a pipette, 2 mL of 20-mM TBA solution and 2 mL filtrated solution were transferred into a test tube and also made a blank sample by the addition of 2 mL distilled water to 2 mL of 20-mM TBA solution. Then, all solutions were carefully stored at room temperature for 15 h. Finally, the concentration of TBARS was evaluated by using a spectrophotometer (Cary 60 UV-Vis, Agilent Technologies, Seoul, Korea) where the reading of absorbance was taken at 531 nm. All samples were arranged in triplicate for analysis.

#### 2.6.4. Determination of Sulfhydryl Groups (Thiol Content)

Total thiol content in cooked beef meatballs was analyzed by following [19] with some modifications to determine the protein oxidation level. In our study, a high-speed homogenizer was installed for homogenization of 2 g ground sample with 25 mL 1% sodium dodecyl sulfate (SDS) in 0.1-M Tris buffer (pH 8.0). The homogenates were allowed to remain in a water bath at 80 °C for 30 min. The test tubes containing homogenates were cooled at room temperature and sent for centrifugation at 7000 *g* for 20 min. After centrifugation, the top layer was strained using an aspirator (A-1000S, Eyela, Japan). Then, filtration was done using Whatman no. 4 filter papers. Then, 0.5 mL filtered solution was transferred in a test tube for mixing with 2 mL 0.1-M Tris buffer (pH 8.0) and 0.5 mL 10-mM DTNB [5,5′-dithiobis (2- nitrobenzoic acid)] in 0.1-M Tris buffer. By following this, a blank sample was made by the combination of 0.5 mL 1% SDS in Tris buffer, 0.5 mL of 10-mM DTNB (5,5′-dithiobis (2-nitrobenzoic acid)) and 2.0 mL 0.1-M Tris buffer. All prepared samples were shaken thoroughly and preserved in a dark room for 30 min. A UV-Vis spectrophotometer (Cary 60, Agilent Technologies, Inc., Seoul, Korea) was used to take the absorbance for thiol concentration at 412 nm and protein content at 280 nm following BSA standard curve. The result was expressed as nmol thiols/mg protein and calculated following the formula:

*Molecular extinction coefficient (E_412_) = 14,000* M^−1^ cm^−1^ (3)

#### 2.6.5. Instrumental Color Evaluation

A colorimeter (CR 300, Minolta, Tokyo, Japan) equipped with a measuring head (CR-300) was used at four different random points on the exterior surface to measure the color qualities of cooked beef meatballs. The color values such as lightness (L^*^), redness (a^*^), yellowness (b^*^), chroma (C^*^), and hue angle (*h°*) values were evaluated in this study. The colorimeter was calibrated using the Standard white plate (Y = 89.2; x = 0.921; y = 0.783) under CIE (Commission international de l’eclairage) illumination D65 and d/10° illumination at room temperature before starting the colorimetry performance of the meatballs. The chroma and hue angle values were calculated applying the equations as follows:

(4) $$Chroma=\sqrt{a^{*2}+b^{*2}}$$

(5) $$Hue\;angle={tan}^{-1}(\frac{b^{*}}{a^{*}})$$

#### 2.6.6. Texture Profile Analysis (TPA)

A digital texture analyzer (EZ-SX, Shimadzu Co., Kyoto, Japan) connected with a computer was used to figure out the subsequent characters like hardness, cohesiveness, springiness, gumminess, chewiness and adhesiveness of the precooked and reheated beef meatballs for TPA. For precooked meatballs, the samples were kept at room temperature for cooling about half an hour after convection-oven cooking, and then each meatball placed on the center of the compression plate with a power cell that was used for measuring the texture followed by the method of [20]. Following this, the reheated meatballs were cooled at room temperature for 30 min after reheating by four different cooking methods separately and placed on the center of the compression plate with a power cell before texture analysis. Force versus time curves was obtained with a 500-N load cell applied at a crosshead speed of 100 mm/min. Two continuous compressions with 1 s interval were applied on (3 × 3 × 3 cm) meatballs. The calculated result of TPA was found by graphing a curve using force and time plots. The mean value of four reproducible runs for each treatment per batch was used in the results section.

### 2.7. Descriptive Sensory Evaluation

#### 2.7.1. Reheating Treatment

Antioxidant-treated meatballs were evaluated for WOF after reheating by boiling, pan-roasting, convection-oven and microwave-oven cooking separately. A detailed description of reheating methods is discussed in Section 2.5. After reheating, the samples were cooled at room temperature for half an hour and kept in a plastic container before presenting to the panelist members.

#### 2.7.2. Selection and Training of Panel Members to Evaluate WOF

A panel consisting of twelve members (aged from 25 to 50 years) was selected from the staff and students of Gyeongsang National University prior to a sensory test. The panelists were experienced in distinguishing original odors and tastes of standard cooked meat products for evaluation of WOF item by temporary ingestion. The training of the panel members was accomplished in four sessions by the roundtable discussion and served a subset of cooked samples to understand the freshly cooked beefy flavor and off-flavor from fat oxidation of precooked meatballs. A brief explanation about the individual sensory parameter with the chosen score was clearly described to each panel member, and the member practiced in that roughly before the final experiment.

#### 2.7.3. WOF Profiling

A total of sixteen (4 treatments × 4 cooking condition) samples were presented to the panel for evaluation at each day. Just after cooling, the reheated samples with some snacks and cool water were presented to the panelists in a randomized order during each session at room temperature. In total 1536 objects (2-month storage × 4 batch × 4 treatments × 4 reheating condition × 12-panel members) were tested for each of the three basic qualities like flavor, taste and overall acceptability of sensory attributes with the preselected score in eight sessions (2 months × 4 batches). For this purpose, the flavor was perceived by the nose to understand fresh beefy odor (score max. 9 for good and min. 5 for bad) or off-odor (score max. 6 for bad and min. 3 for good), the oral taste was perceived by chewing to know the intensity of chewiness (score max. 9 for good and min. 5 for bad), the tongue was used to perceive burnt feelings (score min. 3 for good and max. 6 for bad) and saltiness (score min. 3 for good and 6 for bad), and then overall acceptability was determined lastly by the combination of visual, oral and nasal senses (score max. 9 for good and 5 for bad) to evaluate the WOF of reheated beef meatballs at the end of each month of frozen storage.

### 2.8. Statistical Analysis

All samples measured in this study consist of the observations like 4 treatments × 4 batches × 4 cooking methods × 2 storage periods. All data with four replicated mean values were calculated statistically using the software of Statistical Analysis Systems (SAS^®^ program, SAS version 9.3, SAS Institute, Inc., USA, 2014). A MIXED Model Analysis was implemented to observe the effects of treatment, reheating methods and storage periods on physicochemical characteristics, color, texture and sensory attributes. The three measured variables were considered: dependent variables for physicochemical characteristics, color, texture and sensory attributes; independent variables for treatments, cooking methods and storage times; and random variables for replicates. Least square means for all characteristics were separated (*p* < 0.05) by using the least significant differences (LSMEANS option) produced by the probability difference procedure (PDIFF option).

## 3. Results and Discussion

### 3.1. The Qualitative Content of Chemical Compounds in Nutmeg Extract by UPLC-MS/MS Analysis

**Figure 2:**
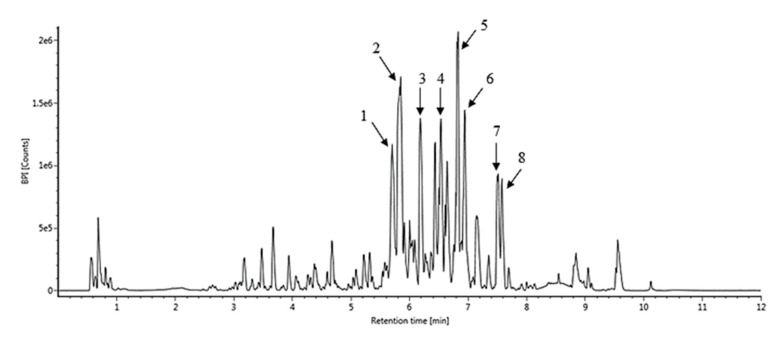
Results of chromatogram after UPLC-MS/MS analysis of chemical compounds of nutmeg extract.

The UPLC-MS/MS analysis of the ethanolic extract of nutmeg is shown in Table 1 and Figure 2. The molecular peak (base peak) at *m/z* 357.16 is in good agreement with the compound (-) -3-(4′-allyl-2′,6′-dimethoxy-phenyloxy)-1-methyl-5-methoxy-1,2-dihydrobenzofuran (1). The compound showed a molecular peak (base peak) at *m/z* 327.15 is good agreement with the derived compound of licarin A (2). The fragmentation showed a peak at *m/z* 325.14 and it showed the presence of compound glabridin (3). The mass spectrum showed a molecular peak (base peak) at *m/z* 359.18 is good agreement with malabaricone C (4). The fragmentation showed a peak at *m/z* 357.17 and it indicated the presence of 5-methoxylicarin A (5). The compound showed a molecular peak (base peak) at *m/z* 327.15 is exactly in agreement with licarin A (6).

The fragmentation also showed the exact value at *m/z* 355.15, so it revealed the presence of 5-methoxylicarin B (7). The compound showed a molecular peak (base peak) at *m/z* 325.14 is good agreement with the licarin B (8). [21] found that the main bioactive compounds as lignans isolated from *M. fragrans* seeds were erythro-austrobailignan-6, meso-dihydroguaiaretic acid and nectandrin-B together with macelignan, machilin F, nectandrin B, licarin A, licarin B, myristagenol and mesodihydroguaiaretic acid which had antioxidant and phytochemical properties.

### 3.2. Physicochemical Properties of Precooked Beef Meatballs

The effect of precooking by convection oven on the physicochemical characteristics of antioxidants added beef meatballs is presented in Table 2. The pH values of all treatments showed a non-significant *(p* > 0.05) difference after precooked by convection oven. Hence, the addition of BHT, nutmeg extract, and nutmeg powder had no effect on the pH of cooked meatballs. The findings are in agreement with [22] as BHT/BHA in beef burger had no effect on pH and also [23], reported that the pH of ground beef burgers showed no significant change after the application of the poppy seeds into samples. Water and fat retention capacity of meat products determine the volume of cooking loss during thermal treatment [24]. All antioxidants treated samples and control after convection-oven cooking showed statistically insignificant difference (*p* > 0.05) in cooking loss.

The color of meat is a vital quality of meat product consumption or rejection to the customers [25]. The color attributes were remaining unchangeable in all antioxidant-added meatballs along with control after precooked by convection oven. The color values did not change significantly in BHT-, nutmeg extract- and nutmeg-powder-added meatballs (*p* > 0.05). The obtained results declared the similar effect of nutmeg on maintaining the redness color of meat products as given by [14]. Moreover, [26] recorded that the formation of bright redness and decreased brown color in meatballs could be achieved by the action of natural antioxidants.

The antioxidant free control samples had significantly higher (*p* < 0.05) TBARS values than BHT-, nutmeg extract- and nutmeg-powder-added meatballs. The TBARS value of nutmeg-extract-added meatballs was significantly reduced (*p* < 0.05) when compared to other meatballs and M1 and M3 showed statistically insignificant (*p* > 0.05) effect on reducing TBARS value. The result of the effectiveness of nutmeg extracts is in agreement with earlier studies that the application of nutmeg extracts substantially delayed the raise of TBA values in raw meat and cooked sausages [27]. Protein oxidation or thiol oxidation results in loss of protein solubility which is responsible for the deterioration of standard meat texture and tenderness. No significant differences in thiol contents were found among all meatballs samples when precooked by convection oven (*p* > 0.05). The application of BHT, nutmeg extracts and nutmeg powder in meatballs reported statistically insignificant effect (*p* > 0.05) in inhibiting protein solubility and protein oxidation by building a cluster of protein through internal cross-linkage to hamper enzymatic proteolysis during convection-oven cooking for meatballs [28].

The myofibrillar protein molecules, soluble protein and connective tissue of raw meat sample undergoes a random transformation during cooking and this phenomenon is evaluated by means of hardness, springiness, cohesiveness, gumminess, chewiness and adhesiveness to know the changes in muscle protein functionality and non-meat ingredients of raw meat during texture analysis. The hardness of BHT-, nutmeg extract- and nutmeg-powder-formulated meatballs was significantly reduced (*p* < 0.05) after cooked by convection oven when compared with the control. This may have occurred because of the inhibitory effect of antioxidants against the increased hardness of cooked meatballs. Antioxidants act as a barrier for lipid oxidation, loss of moisture and conversion of texture by upholding the integrity of muscle membrane in meat products [29]. On the other hand, the convection-oven cooking had no significant effect (*p* > 0.05) on cohesiveness, springiness, gumminess, chewiness and adhesiveness of the control and other antioxidants-treated meatballs samples. The findings are in accordance with those of [30], who reported that antioxidants had no textural effect on cohesiveness, springiness, gumminess and chewiness in fish sausages at 0-day storage.

### 3.3. Measurement of Lipid Oxidation of Reheated Beef Meatballs

**Figure 3:**
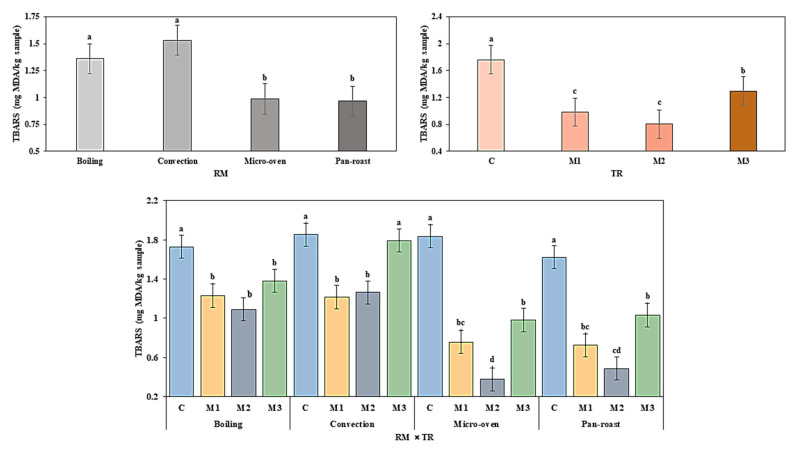
Effect of different reheating condition on thiobarbituric acid-reactive substances (TBARS) of nutmeg extract enriched precooked beef meatballs during frozen storage. RM, reheating method; ST, storage time; C, antioxidant-free meatballs; M1, 0.02% BHT treated; M2, 0.02% nutmeg extract treated; M3, 0.02% nutmeg powder treated. ^a–d^ Means with different superscript small letters differ significantly (*p* < 0.05) in TBARS among treatments at different reheating method.

The thiobarbituric acid (TBA) test is a reduction reaction against TBA and malonaldehyde, which identify the oxidative state of animal fat. Lipid oxidation levels of different reheated meatballs during frozen storage are presented in Figure 3.

Both BHT as a synthetic antioxidant and nutmeg extract as a natural antioxidant had shown noticeable antioxidant activities in preventing lipid oxidation in our investigation. A significantly (*p* < 0.05) higher TBARS values were found for the control when compared to other antioxidants-added meatballs after reheating. The secondary product of lipid oxidation is MDA which is responsible for the production of higher TBARS values of control in beef patties [31]. The BHT and nutmeg-extract-added meatballs had significantly (*p* < 0.05) lower TBARS values than the control and nutmeg-powder-added meatballs during frozen storage; nevertheless, statistically insignificant (*p* > 0.05) difference in TBARS values were found between BHT (0.98 mg MDA/kg sample) and nutmeg extract (0.80 mg MDA/kg sample)-added meatballs after reheating. Here, BHT and nutmeg extracts were functioning as equal restraint for lipid oxidation. Reheated meatballs by pan-roasting and microwave oven indicated significantly (*p* < 0.05) lower TBARS values than boiling and convection-oven cooking and these results agree with the outcome of Choi (2008) [8]. Cooking at high-temperature prior to consumption extends TBARS values by accelerating the lipid oxidation process as reported by [32]. The interaction of cooking methods and types of treatment showed significant differences (*p* < 0.05) in TBARS values among meatballs samples after application of different reheating methods. The control sample of boiling, microwave-oven and pan-roasting cooking method presented significantly (*p* < 0.05) higher TBARS values as compared with BHT-, nutmeg extract-s and nutmeg-powder-added meatballs. After convection oven reheating, BHT and nutmeg-extract-added meatballs had significantly (*p* < 0.05) decreased TBARS values when compared to the control and nutmeg-powder-containing meatballs. The meatballs with added nutmeg extract had significantly (*p* > 0.05) lower rate for lipid oxidation than all other meatballs samples after microwave oven reheating. These findings are in accordance with [33], who indicated that natural antioxidant-containing restructured beef steaks exhibited lower lipid oxidation rate for application of different cooking methods.

### 3.4. Measurement of Protein Oxidation of Reheated Beef Meatballs

**Figure 4:**
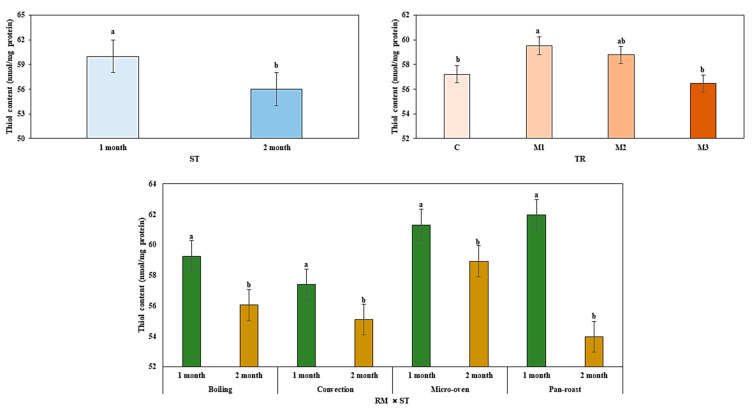
Effect of different reheating condition on thiol content of nutmeg extract enriched precooked beef meatballs during frozen storage. RM, reheating method; ST, storage time; C, antioxidant-free meatballs; M1, 0.02% BHT treated; M2, 0.02% nutmeg extract treated; M3, 0.02% nutmeg powder treated. ^a–b^ Means with different superscript small letters differ significantly (*p* < 0.05) in thiol content among treatments at different reheating method within 1 and 2 months independently.

The oxidation of protein causes the destruction of protein structure and loss of properties in meat products, which is determined by thiol content and the level of free thiols quantified by DTNB. The thiol content of reheated meatballs was greatly affected by the type of treatment and storage time in our study presented in Figure 4.

The thiol concentration ranged from 57.38 to 61.96 (nmol/mg protein) in all reheated meatballs. The thiol content of beef meatballs was significantly reduced (*p* < 0.05) from 1st month to 2nd month of frozen storage, implied the oxidation of protein with rising of storage time. The BHT-added meatballs as M1 had significantly (*p* < 0.05) higher values for thiol content when compared to the control and M3; however, a statistically insignificant difference (*p* > 0.05) was seen for thiol content of M1 and M2. The nutmeg-extract-containing meatballs showed insignificantly (*p* > 0.05) higher thiol content than the control and nutmeg-powder-added meatballs. Therefore, our data proved that nutmeg extract as a natural antioxidant performed an identical role as a synthetic antioxidant of BHT in the reduction of protein oxidation. Increased protein oxidation is related to the disintegration of free thiol groups of disulfide bond formation. Furthermore, a similar effect on the reduction of protein oxidation by the inclusion of plant extract as a natural antioxidant in meat products has been indicated [34]. The formation of lower TBARS values is associated with the higher thiol content by the addition of antioxidants to meat products [35], indicated that low level of lipid and protein oxidation occurred in M1 and M2. Moreover, the interaction between cooking systems with storage periods significantly (*p* < 0.05) increased protein oxidation by reducing thiol content while extending storage period. The utilization of different reheating methods was significantly (*p* < 0.05) reduced the thiol content of antioxidant-added meatballs from 1st month to 2nd month of frozen storage.

### 3.5. Assessment of Reheating Loss and Color Profile of Reheated Beef Meatballs

Table 3 shows the effect of different reheating methods on reheating loss and the modifications of the color of nutmeg extract enriched beef meatballs after one and two-month of frozen storage at −18 °C.

The addition of antioxidants had no effect on reheating loss of reheated beef meatballs. The reheating loss in meatballs was significantly (*p* < 0.05) increased from convection-oven cooking when compared to other reheating methods. The boiling and microwave oven reheating methods showed significantly (*p* < 0.05) lower reheating loss for meatballs than pan-roasting and convection oven reheating; however, no significant difference (*p* > 0.05) in reheating loss was shown for boiling and microwave oven methods. The variation in the heat transfer rate of individual cooking technique results in fluctuation of heating loss was reported by [36]. The results of our study are in agreement and also disagreement with [37], who observed that the boiling and convection-oven cooking of chicken steak had the maximum cooking loss. In case of reheating loss in meatballs, statistically insignificant (*p* > 0.05) impacts were shown for all antioxidant added treatments and storage periods. Likewise, in the present study, no effects on the cooking loss of meat products were seen for the frozen storage [38]. Moreover, the different reheating methods showed significant (*p* < 0.05) interaction with frozen storage in case of reheating loss.

Quality of meat product and buyer preferences were judged by perceiving the appeared meat color. Here, the addition of antioxidant, four reheating methods and 2 months of frozen storage had a statistically insignificant effect (*p* > 0.05) in the lightness (L^*^) values of color attributes. The lightness values for cooked meat products presented no significant (*p* > 0.05) difference among different cooking methods was also reported by [39]. In this study, antioxidants-added meatballs with the control and frozen storage times showed no significant (*p* > 0.05) change of redness (a^*^) values. In a study by [40] also recorded that frozen storage revealed no significant effects on the color values in beef patties. Nevertheless, the redness (a^*^) values for pan-roasting and microwave oven reheated meatballs was significantly higher (*p* < 0.05) when compared with the methods for boiling and convection oven. The red color is a primary visual aspect of selecting fresh meat products for consumption which is changed because of the cooking methods indicated by [41]. The statistical result pointed out that cooking conditions significantly (*p* < 0.05) denatured myoglobin cells by ice crystallization during frozen storage [42]. In the case of yellowness (b^*^) values, no significant (*p* > 0.05) changes were shown for all antioxidants-treated meatballs with the control, storage periods and reheating methods. After the 2-months of frozen storage, reheated beef meatballs exhibited significantly increased (*p* < 0.05) chroma (C^*^) values and hue angle (*h°*) values. Nonetheless, the *h°* values for pan-roasting and microwave oven reheated meatballs were significantly lower (*p* < 0.05) than the methods for boiling and convection oven. Moreover, the interaction of treatments for reheating methods presented significant (*p* < 0.05) difference between L^*^ and *h°* values in meatballs. The reheating methods exhibited significant (*p* < 0.05) interaction with frozen storage in case of a^*^ and *h°* values of reheated beef meatballs. However, the types of treatments had no significant effect (*p* > 0.05) on any color parameter while interacting with storage period. The results are in accordance with [43], who notified that lightness, redness, yellowness, and chroma values for the control exhibited no significant differences (*p* > 0.05) as compared with the antioxidants treated cooked beef patties.

### 3.6. Evaluation of Instrumental Textural Attributes of Reheated Beef Meatballs

The effects of four different reheating methods of the outcomes of instrumental texture parameters of natural and synthetic antioxidant added precooked beef meatballs after two-month frozen storage are shown in Table 4. Major requirements of meat products consumption are textural properties identified by the degree of extraction of myofibrillar protein, stromal protein, comminution degree and non-meat ingredients [44].

Hardness is the key element of the textural profiles which signify the market worth of meat items. In our study, the antioxidants supplemented precooked meatballs exhibited significantly (*p* < 0.05) reduced hardness values before reheating (Table 2), whereas statistically insignificant (*p* > 0.05) effect of hardness values were found for antioxidants enriched reheated meatballs. The antioxidants-added meatballs and the control showed no significant (*p* > 0.05) changes in all textural attributes after reheating. The reheating methods of boiling and convection oven had significantly higher (*p* < 0.05) springiness in meatballs than the methods of pan-roasting and microwave oven. However, reheating of meatballs by convection oven had significantly (*p* < 0.05) higher gumminess and lower adhesiveness, respectively, when compared to all other methods. Furthermore [45] reported that different cooking methods had no significant difference (*p* > 0.05) for stringiness, gumminess and resilience except hardness, but oven cooking showed significant difference (*p* < 0.05) for cohesiveness and chewiness values of all chicken meat cutlets. The use of ingredients and preparation of batter were basic determiners for meat product texture quality. The texture attributes of hardness, springiness, gumminess, and chewiness were numerically lower (*p* > 0.05) for the control when compared to antioxidants-added meatballs after reheating, since the rigid structure of plants extract-treated meat products developed because of the increase of protein level [46]. Similarly, reheated meatballs by boiling showed significantly (*p* < 0.05) higher springiness and adhesiveness. In our results, boiling and oven reheated meatballs contained higher levels of moisture, and accordingly springiness is interrelated with the water content of meat products and adhesiveness also indicates the presence of higher moisture at the surface of meat products. From month 1 to month 2 of frozen storage, the gumminess for reheated meatballs was significantly decreased (*p* < 0.05), but a statistically insignificant (*p* > 0.05) change was seen for all other texture attributes. Moreover, the interaction for treatments and reheating methods, treatments and storage, and reheating methods and storage were significantly (*p* < 0.05) different for adhesiveness value, while all texture parameters except adhesiveness showed statistically insignificant (*p* > 0.05) difference for all interactions.

### 3.7. WOF Evaluation of Reheated Beef Meatballs

The effect of different reheating methods and frozen storage on the sensory attributes of nutmeg extracts enriched precooked beef meatballs were evaluated as WOF and are presented in Table 5. At the beginning of the investigation, the consequence of natural antioxidants on meatballs was easily recognized, and panel taste scores for fresh beefy flavor were not significantly (*p* > 0.05) different for all meatball samples after reheating. This means the inclusion of nutmeg extracts in meatballs had no negative impact on fresh beefy flavor, which was similar to [47]. The control meatballs had significantly higher (*p* < 0.05) rancid flavor than the nutmeg extract- and powder-added meatballs after reheating. Pan-roasting showed significantly the highest (*p* < 0.05) rancid flavor for reheated meatballs; the rancid flavor for all meatballs significantly (*p* < 0.05) increased after 1st month to 2nd months of frozen storage. Rancidity is a worse criterion of meat product which develops unpleasant flavor from lipid oxidation after long term storage [48]. The chewiness value of the reheated control sample was significantly (*p* < 0.05) lower among antioxidants-added meatballs. However, a significant increase (*p* < 0.05) in the score of the intensity of the chewiness was found along with low rancid flavor for all antioxidants added sausages [30]. Again, reheating by boiling had significantly higher (*p* < 0.05) chewiness intensity when compared to pan-roasting and convection oven reheated meatballs. The addition of antioxidants to reheated meatballs significantly increased (*p* < 0.05) the chewiness intensity from month 1 to month 2 of frozen storage. The burnt tastes and saltiness for nutmeg-extract-containing meatballs were significantly (*p* < 0.05) lower when compared to all other meatballs after reheating. Among the four reheating methods, pan-roasting showed significantly the highest (*p* < 0.05) level of burnt taste for meatballs. It is observed that higher level of burnt taste was reflective of higher level of rancid flavor because of the oxidative changes. The overall acceptability was significantly increased (*p* < 0.05) for M1 and M2 as compared with the control and M3. However, the different reheating methods had no effect on the overall acceptability of reheated meatballs. The overall acceptability for reheated meatballs was significantly (*p* < 0.05) higher for 1st month than 2nd month, indicated the reduction of acceptability with the increase in frozen storage time.

The interaction for the cooking system and storage was significantly (*p* < 0.05) different for chewiness strength, saltiness and overall acceptability, which was accelerated by the existence of fat and lipid in the final product [49]. Likewise, the chewiness intensity and burnt taste for meatballs were also significantly (*p* < 0.05) influenced by the interaction of treatment and frozen storage. Nonetheless, the interaction of treatments and reheating methods showed insignificant effect (*p* > 0.05) on all sensorial attributes. After all, the high intensity of chewiness with low rancidity, burnt taste and saltiness for 0.02%-nutmeg-extract-containing meatballs resulted in most panel acceptability, and the reheating methods of boiling and microwave oven led to a higher chewiness and a lower level of rancidity and burnt taste. There were no noticeable changes in fresh beefy flavor and rancid flavor development during interaction concerning the type of meatballs, reheating methods and frozen storage.

## 4. Conclusions

The addition of antioxidants in fresh beef meatballs showed no significant (*p* > 0.05) influence on the basic physicochemical characteristics except for increasing oxidative (lipid and protein) stability and reducing hardness value of texture after precooking using a convection oven method. After two months of frozen storage, 0.02%-nutmeg-extract–treated meatballs reheated by microwave oven method significantly (*p* < 0.05) reduced the oxidation rate, reheating loss and WOF development in terms of keeping meat color stable, increasing chewiness intensity and overall acceptability by delaying rancidity. On the other hand, convection oven as a reheating method negatively influenced the product qualities by extending cooking loss, lipid oxidation, redness, hue angle, gumminess, adhesiveness and springiness of meatballs when compared to other cooking methods. The nutmeg extracts effectively retarded the formation of rancid off-flavor of precooked beef meatballs during frozen storage. Therefore, this study indicated the addition of nutmeg extract as a natural antioxidant instead of BHT to beef meatballs could effectively inhibit lipid and protein oxidation as well as to exhibit positive effects on color, texture and WOF of precooked meatballs during frozen storage and microwave-oven reheating at the industrial levels and small scale levels.

## Figures and Tables

**Table 1:** List of chemical compounds identified from UPLC-MS/MS results of nutmeg extract.

Component Name	Observed RT (min)	Observed *m/z*	Fragment	Adducts
1. 3-(4′-allyl-2′,6′-dimethoxy-phenyloxy)-1-Methyl-5-methoxy-1,2-dihydrobenzofuran	5.71	357.1697	251, 221, 203, 181, 167, 151, 137	+H
2. Licarin A-derived compound	5.83	327.1592	298, 203, 188, 165	+H
3. Glabridin	6.19	325.1437	310, 293, 193, 115	+H
4. Malabaricone C	6.54	359.1855	249, 231, 163, 123	+H
5. 5-Methoxylicarin A	6.82	357.1700	325, 188	+H
6. Licarin A	6.94	327.1592	203, 188	+H
7. 5-Methoxylicarin B	7.51	355.1539	188	+H
8. Licarin B	7.58	325.1444	203, 188	+H

**Table 2:** Effect of precooking (convection oven) on physicochemical characteristics of nutmeg extract enriched beef meatballs.

Items		Treatments				SEM
		Control ^1^	M1	M2	M3	
pH		6.06	6.04	6.07	6.05	0.06
Cooking loss (%)		15.28	16.07	14.52	14.77	1.17
Color	Lightness (L^*^)	50.59	49.86	50.27	51.34	0.89
	Redness (a^*^)	8.69	9.14	9.12	8.77	0.45
	Yellowness (b^*^)	13.04	13.01	13.01	12.87	0.39
	Chroma (C*)	15.68	15.90	15.90	15.58	0.57
	Hue angle (*h°*)	56.37	54.96	55.07	55.78	0.63
TBARS (mg MDA/kg sample)		0.99 ^a^	0.51 ^b^	0.23 ^c^	0.53 ^b^	0.11
Thiol content (nmol/mg protein)		68.72	68.53	73.57	66.21	2.71
Texture profile analysis (TPA)	Hardness (N)	13.67 ^a^	11.93 ^b^	11.59 ^b^	10.45 ^b^	0.69
	Cohesiveness	0.42	0.43	0.42	0.47	0.02
	Springiness (mm)	1.14	1.07	1.11	1.31	0.084
	Gumminess (N)	5.76	5.06	4.85	4.91	0.43
	Chewiness (N*mm)	6.53	5.44	5.45	6.49	0.52
	Adhesiveness	−0.03	−0.03	−0.03	−0.02	0.005

^1^ Control—antioxidant-free meatballs; M1—0.02% BHT–treated meatballs; M2—0.02% nutmeg extract–treated meatballs; M3—0.02% nutmeg powder-treated meatballs. ^a–c^ Means in the same row with different superscripts are significantly different (*p* < 0.05). SEM are standard error of the mean values of three determination.

**Table 3:** Effect of different reheating condition on reheating loss and color parameters of nutmeg-extract-added beef meatballs during frozen storage.

Effects	Items	Reheating Loss (%)	Lightness (L^*^)	Redness (a^*^)	Yellowness (b^*^)	Chroma (C^*^)	Hue Angle (*h°*)
Treatments (TR)	Control ^1^	7.31	38.28	8.22	15.01	16.88	61.56
	M1	6.42	39.14	8.61	14.32	16.92	59.24
	M2	6.83	38.28	8.49	14.55	16.88	59.69
	M3	6.21	38.75	8.40	14.18	16.91	59.90
SEM		0.69	1.06	0.16	0.40	0.30	0.95
Reheating method (RM)	Boiling	2.62 ^c^	40.59	8.02 ^b^	15.14	17.10	62.24 ^a^
	Pan-roasting	8.12 ^b^	35.92	9.38 ^a^	14.29	17.22	57.32 ^b^
	Convection oven	13.38 ^a^	34.96	7.50 ^b^	14.18	16.05	62.27 ^a^
	Microwave oven	2.64 ^c^	42.98	8.82 ^a^	14.45	17.21	58.58 ^b^
SEM		0.69	0.91	0.26	0.37	0.33	0.92
Storage time (S)	1 month	7.09	38.57	8.52	14.02	16.48 ^b^	58.79 ^b^
	2 month	6.29	38.57	8.34	15.01	17.31 ^a^	61.41 ^a^
SEM		0.398	0.86	0.19	0.38	0.24	0.65
Interaction	TR * RM	0.99	0.04	0.22	0.95	0.95	0.08
	TR * S	0.91	0.73	0.39	0.98	0.21	0.82
	RM * S	0.04	0.73	0.02	0.98	0.64	0.003

^1^ Control, antioxidant-free meatballs; M1, 0.02% BHT treated meatballs; M2, 0.02% nutmeg extract treated meatballs; M3, 0.02% nutmeg powder treated meatballs. TR, RM and S were used to express the treatment, reheating method and storage time. ^a–c^ Means in the same row with different superscripts are significantly different (*p* < 0.05). SEM are standard error of the mean values of three determination.

**Table 4:** Effect of different reheating condition on the texture profile analysis of nutmeg extract enriched beef meatballs during frozen storage.

Effects	Items	Attributes					
		Hardness (N)	Cohesiveness	Springiness (mm)	Gumminess (N)	Chewiness (N*mm)	Adhesiveness
Treatments (TR)	Control ^1^	9.85	0.46	1.53	4.48	6.83	−0.02
	M1	10.20	0.48	1.57	4.84	8.05	−0.03
	M2	10.08	0.49	1.62	4.88	8.09	−0.04
	M3	10.18	0.47	1.54	4.70	7.59	−0.03
SEM		0.62	0.01	0.16	0.27	1.01	0.01
Reheating method (RM)	Boiling	10.01	0.47	1.85 ^a^	4.55 ^b^	8.54	−0.03 ^a^
	Pan-roasting	9.78	0.46	1.34 ^b^	4.67 ^b^	6.43	−0.02 ^a^
	Convection oven	12.09	0.47	1.82 ^a^	5.57 ^a^	10.71	−0.04 ^b^
	Microwave oven	8.42	0.48	1.25 ^b^	4.12 ^b^	4.88	−0.02 ^a^
SEM		0.76	0.02	0.11	0.304	1.28	0.01
Storage time (S)	1 month	10.35	0.49	1.54	5.09 ^a^	8.13	−0.03
	2 month	9.81	0.46	1.58	4.37 ^b^	7.15	−0.03
SEM		0.27	0.02	0.02	0.36	0.49	0.001
Interaction	TR * RM	0.87	0.39	0.98	0.57	0.90	0.02
	TR * S	0.87	0.59	0.95	0.94	0.81	0.04
	RM * S	0.70	0.41	0.44	0.72	0.88	0.09

^1^ Control, antioxidant-free meatballs; M1, 0.02% BHT treated meatballs; M2, 0.02% nutmeg extract treated meatballs; M3, 0.02% nutmeg powder treated meatballs. TR, RM and S were used to express the treatment, reheating method and storage time. ^a,b^ Means in the same row with different superscripts are significantly different (*p* < 0.05). SEM are standard error of the mean values of three determination.

**Table 5:** Effect of different reheating condition on the warmed-over flavor of nutmeg extract enriched beef meatballs during frozen storage.

Effect	Items	Flavor		Taste			Overall Acceptability
		Fresh Beefy	Rancid	Intensity of Chewiness	Burnt Taste	Saltiness	
Treatments (TR)	Control ^1^	6.11	3.49 ^a^	6.21 ^b^	3.39 ^a^	3.34 ^a^	6.09 ^b^
	M1	6.38	3.41 ^ab^	6.57 ^a^	3.37 ^a^	3.39 ^a^	6.61 ^a^
	M2	6.28	3.30 ^b^	6.86 ^a^	3.19 ^b^	3.22 ^b^	6.67 ^a^
	M3	5.92	3.33 ^b^	6.65 ^a^	3.36 ^a^	3.36 ^a^	6.09 ^b^
SEM		0.12	0.05	0.11	0.05	0.06	0.13
Reheating method (RM)	Boiling	6.35	3.34 ^b^	6.87 ^a^	3.17 ^bc^	3.22	6.56
	Pan-roasting	6.18	3.47 ^a^	6.41 ^b^	3.66 ^a^	3.44	6.45
	Convection oven	6.07	3.36 ^b^	6.40 ^b^	3.36 ^b^	3.43	6.26
	Microwave oven	6.09	3.37 ^b^	6.61 ^ab^	3.12 ^c^	3.21	6.19
SEM		0.14	0.05	0.11	0.08	0.10	0.13
Storage time (S)	1 month	6.11	3.25 ^b^	6.25 ^b^	3.29	3.26	6.69 ^a^
	2 month	6.23	3.52 ^a^	6.89 ^a^	3.35	3.39	6.04 ^b^
SEM		0.12	0.06	0.08	0.05	0.07	0.09
Interaction	TR * RM	0.39	0.99	0.76	0.17	0.91	0.96
	TR * S	0.94	0.38	0.02	0.03	0.93	0.14
	RM * S	0.39	0.24	<0.0001	0.74	0.01	0.001

^1^ Control, antioxidant-free meatballs; M1, 0.02% BHT treated meatballs; M2, 0.02% nutmeg extract treated meatballs; M3, 0.02% nutmeg powder treated meatballs. TR, RM and S were used to express the treatment, reheating method and storage time. ^a–c^ Means in the same row with different superscripts are significantly different (*p* < 0.05). SEM are standard error of the mean values of three determination.
